# Mental health literacy: a comparative study on stigmatizing attitude and help-seeking behavior towards mental disorders between adolescents and adults

**DOI:** 10.1186/s42506-025-00184-0

**Published:** 2025-04-28

**Authors:** Amal M. I. Goda, Salwa A. Abd Elhamid, Ghada O. Wassif

**Affiliations:** 1 Department of Community, Environmental and Occupational Medicine, Faculty of Medicine, Ain Shams University, Cairo, Egypt; 2 Department of Pediatrics, Faculty of Medicine, Ain Shams University, Cairo, Egypt

**Keywords:** Mental health illnesses, Mental health literacy, Stigmatizing attitude

## Abstract

**Background:**

Mental health awareness has the potential to contribute to the prompt identification and effective management of mental disorders. The negative perception connected to mental conditions presents a significant challenge for individuals seeking mental health services and the professionals providing them. This study aims to compare mental health literacy—encompassing knowledge, attitudes, and behaviors—between adults and adolescents attending Ain Shams University Hospitals’ outpatient clinics and identify the socio-demographic factors that could predict these components.

**Methods:**

The present survey was carried out during the years 2022–2023 on 369 individuals, who were 11 years or older. An Arabic questionnaire, validated and tested for reliability, was employed to evaluate knowledge, attitudes, and behaviors related to mental disorders. The tools consisted of three validated scales: the Mental Health Knowledge Schedule (MAKS), the Community Attitudes towards Mental Illness (CAMI), and the Reported and Intended Behavior Scale (RIBS).

**Results:**

Adults demonstrated significantly higher knowledge scores (46.33 ± 4.69 vs. 43.16 ± 4.92, *p* ≤ 0.01) and behavior scores (16.66 ± 3.56 vs. 15.53 ± 3.71, *p* ≤ 0.01), while adolescents exhibited more favorable attitudes (99.03 ± 17.43 vs. 90.74 ± 11.78, *p* ≤ 0.01). Higher knowledge was associated with being female, having a university education, living in urban areas, and being employed. Favorable attitudes were linked to adolescents, males, rural residents, and lower educational levels. At the same time, positive behaviors were associated with being female, having a university education, living in urban areas, being employed, and knowing someone with a mental illness. Regression analysis highlighted education and urban residence as consistent predictors across all mental health literacy components, with employment and familiarity with mental illness further enhancing behavior scores.

**Conclusion:**

This study highlights significant differences in mental health literacy between adults and adolescents, with adults exhibiting higher knowledge and behavior scores and adolescents demonstrating more favorable attitudes. Socio-demographic factors, particularly education, urban residence, and employment, emerged as consistent predictors influencing knowledge, attitudes, and behaviors. These findings underscore the need for targeted interventions, such as incorporating mental health education into curricula, launching stigma-reduction campaigns, and improving access to mental health services, particularly in rural areas.

## Introduction

Adolescents and young adults are the age group most susceptible to mental health illnesses, although these conditions can also begin in childhood. Mental illnesses are considered to be among the main causes of morbidity, mortality, and disability [1]. People who suffer from mental illness often face not only the challenges of their illness but also poverty and the stigma attached to it [2]. Because they have unfavorable opinions of mental health services, they often avoid using them [3].

Mental health illiteracy contributes to the stigma associated with individuals suffering from mental illness. This lack of understanding is widespread, not only in developing countries [4] such as Iran [5] and Turkey [6], but also in developed countries such as the USA [7].

To encourage seeking help and supportive actions within the community, as well as to enhance comprehension of mental health, it is crucial to address the gap in knowledge and negative attitudes towards mental illness among both adolescents and adults [8]. By reducing stigma, we can safeguard the human rights of individuals with mental health conditions [9].

There is an increasing number of research on adult mental illness stigma and strategies for eliminating it. Still, little is known about how adolescents view those who suffer from mental illnesses. Few studies, such as *Elaidy & Arafa, 2020* [10], which examined university students, Tosson et al., 2020 [11], which examined adults, and *Shehata and Abdeldaim, 2020* [12], which examined medical personnel, have been conducted in Egypt to assess knowledge, beliefs, and attitudes regarding mental illness in various age and job strata.

Nevertheless, research comparing the stigmatization associated with mental disorders in adults and adolescents has not yet been conducted. A comparison of the attitudes of adults and adolescents regarding mental illness could aid in the discovery of significant differences and open the door for focused intervention programs designed to raise mental health literacy in populations where attitudes towards mental illness are unfavorable. As a result, a more accepting environment will be created, and patients will be encouraged to seek appropriate mental health services to detect their illness early and receive support for their treatment without any fears. This study aims to compare mental health literacy—encompassing knowledge, attitudes, and behaviors—between adults and adolescents attending Ain Shams University Hospitals’ outpatient clinics and identify the socio-demographic factors that could predict these components.

## Methods

### Study type & population

A cross-sectional study was conducted during 2022–2023 in various outpatient clinics at Ain Shams University Hospitals. The study population included two distinct categories: adolescents, operationally defined as individuals aged 11–19 years, and adults, operationally defined as those aged 20 years and above. These definitions align with the World Health Organization’s age classifications and were adopted to ensure consistency throughout the study [13].

Systematic random sampling was used to ensure that the study results could be generalized and that all patients and their relatives within the hospital were adequately represented. Participants, including adolescents and adults, were selected based on their attendance patterns at outpatient clinics. Recruitment was conducted over two rotating days each week to ensure variability and to capture a diverse sample of individuals attending the hospital. Study participants were recruited from various outpatient clinics, including internal medicine, pediatrics, and psychiatry. This approach was designed to capture a broad representation of hospital attendees while ensuring systematic and unbiased selection.

Data collection was carried out in designated, quiet areas within the outpatient clinics to ensure confidentiality and minimize distractions. Participants were approached in person by the researchers while waiting in the outpatient clinics, and their participation was voluntary. Data collection occurred between 9:00 AM and 3:00 PM, during the clinics’ peak operating hours to ensure the inclusion of a variety of participants.

### Sample size

The sample size was calculated using Open Epi, Version 3, an open-source calculator, and based on a study by *Bragg *et al*., 2018* [14]. A sample size of 369 participants was sufficient to achieve the study objectives based on a hypothesized % frequency of the outcome factor = 60.0% (% of participants with health literacy of mental disorders), confidence level 95%, and margin of error ± 5.

### Study tools

The Arabic version of the questionnaire was validated by *Abi Doumit *et al*., 2019* [3] through a rigorous process of translation and back-translation to ensure linguistic and cultural appropriateness. In this study, face and construct validation were performed by the researchers to ensure the tool’s suitability for the target population. Face validation was carried out by a panel of three experts, including two public health consultants and one psychiatric consultant, who assessed the clarity, relevance, and comprehensibility of each item. The same panel also evaluated construct validity to confirm that the items adequately represented the theoretical constructs being measured, ensuring the questionnaire captured the intended dimensions of the study variables. This combined process ensured the validity and applicability of the instrument for use in the target population.

The questionnaire consists of five sections. The completion of the questionnaire required an average time of 25 min. The first section encompassed the personal and socio-demographic characteristics of the participants.

The second section entailed the Mental Health Knowledge Schedule (MAKS), which comprised twelve questions. This instrument is considered valid, reliable, brief, and feasible for evaluating and monitoring evidence-based knowledge concerning stigma toward mental illness. The Cronbach’s alpha coefficient for the Arabic version of the MAKS scale was 0.749 [3]. The component of the MAKS scale involved two forms of knowledge. The first four questions focused on individuals’ familiarity with different mental health disorders, such as depression and bipolar disorder. This effectively demonstrated how individuals perceive these disorders and whether they view them as conditions requiring professional assistance or care. The second set of inquiries pertains to the bias and segregation directed towards individuals with mental illness, with scoring being conducted on a Likert scale ranging from 1 to 5. Items that the respondent strongly agrees with will receive five points, while items that the respondent strongly disagrees with will receive one point. The total points earned for all 12 items are added up to determine the final score. A higher level of understanding is indicated by higher total scores [15].

The third section encompasses The Community Attitudes towards Mental Illness (CAMI). It consists of forty questions in total; with four subscales, each of which contains 10 items. It has been adapted and modified from the work of *Taylor and Dear, 1981* [16] by *Abi Doumit *et al*., 2019* [3].

The Community Attitudes towards Mental Illness (CAMI) response categories is in the form of a Likert scale that consists of five points, ranging from "strongly agree" to "strongly disagree" on a numerical scale from 1 to 5. Items that were stated in a negative manner were reversed and recorded for the purpose of analysis [3].

The four dimensions that shape perceptions and stigma in this tool are Authoritarianism (AU), which reflects viewing individuals with mental illness as inferior and requiring control or supervision, while Benevolence (BE) represents a compassionate and empathetic perspective toward them. Social Restrictiveness (SR) embodies the belief that people with mental illness pose a societal threat and should be avoided. In contrast, Community Mental Health Ideology (CMHI) emphasizes the acceptance of mental health services and integrating individuals with mental illness into the community. A person will be more stigmatized if scores higher on the Community Mental Health Ideology (CMHI) scale, lower on the Benevolence (BE) scale, higher on the Authoritarianism (AU) scale, and lower on the Social Restrictiveness (SR) scale. Overall stigma is calculated by summing the subscale scores, where higher totals signify more positive attitudes and reduced stigma toward individuals with mental illness. The following are the Cronbach’s alpha coefficients for the overall scale Community Attitudes towards Mental Illness (CAMI) = 0.876 and each of its subscales: Community Mental Health Ideology (CMHI) = 0.804, Benevolence (BE) = 0.637, Social Restrictiveness (SR) = 0.690, Authoritarianism (AU) = 0.555 [3].

The fourth section of the assessment includes the Reported and Intended Behavior Scale (RIBS), which consists of eight questions arranged into two groups. The first set of questions concerns behavior that has been reported in previous or present experiences concerning a variety of topics, including cohabiting, working with, living close to, or developing a relationship with someone who has a mental health problem. The second set of questions focuses on the plans to make future contact in the same areas with people having mental health issues. A Likert scale is used to assign a score to each item, with 1 denoting strongly disagreed and 5 denoting strongly agreed. "Do Not know," the neutral response, is likewise coded neutral. It is significant to remember that more positive expected behaviors are indicated by higher values on the scale [17]. The Reported and Intended Behavior Scale (RIBS) scale’s Cronbach’s alpha coefficient was determined to be 0.766 [3].

### Statistical analysis

The data collected underwent a process of revision, coding, and tabulation, and entered in a personal computer to be analyzed with IBM SPSS version 26.0. The parametric numerical data was subjected to calculations of the mean, standard deviation (SD), and range. The independent sample t-test, chi-square test, One-way ANOVA, and Correlation Coefficient (r) were employed to test statistical significance when appropriately indicated. Multiple linear Regression analysis was employed to determine the independent predictors for Knowledge, attitude, and behavior scores toward mental illness. The P-value served as an indicator of the level of significance where a p-value ≤ 0.05 is considered significant.

## Results

The sociodemographic characteristics of the studied participants show that their mean age was 26.31 ± 10.05 years, ranging from 11 to 70 years. Adolescents (11–19 years) comprised 32.0% of the sample, while adults (20 years and above) accounted for 68.0%. The majority of participants were female (70.2%) and had a university degree (76.4%). Regarding marital status, 61.2% of participants had never been married. Employment data showed that 59.1% of participants were unemployed. Most participants resided in urban areas (86.4%). Additionally, 66.4% of participants reported knowing someone with a mental illness (Table 1).

**Table 1:** Socio-demographic characteristics of the studied participants, Ain Shams University hospitals, 2022–2023 (*n* = 369)

Socio-demographic characteristics	No. (%)
**Age**	Mean ± SD (26.31 ± 10.05) Range (11–70)
Adolescents (11–19 years)	118 (32)
Adults (20 + years)	251 (68)
**Gender**	
Male	110 (29.8)
Female	259 (70.2)
**Educational level**	
Primary	19 (5.1)
Preparatory	22 (6.0)
Secondary	46 (12.5)
University	282 (76.4)
**Marital status**	
Never been married	226 (61.2)
Ever been married^a^	143 (38.8)
**Employment status**	
Do not work	218 (59.1)
Work	151 (40.9)
**Residence**	
Urban	319 (86.4)
Rural	50 (13.6)
**Knowing someone with a mental illness**	
No	124 (33.6)
Yes	245 (66.4)

^a^Married, divorced, and widowed

The association between socio-demographic factors and the mean scores of knowledge, attitude, and behavior toward people with mental health disorders reveal that adults demonstrated significantly higher knowledge and behavior scores (46.33 ± 4.69 and 16.66 ± 3.56, respectively) compared to adolescents (43.16 ± 4.92 and 15.53 ± 3.71, respectively, *p* ≤ 0.01), Conversely, adolescents had a higher total attitude score (99.03 ± 17.43) than adults (90.74 ± 11.78, *p* ≤ 0.01). Females scored higher in knowledge (45.86 ± 4.83 vs. 44.02 ± 5.12, *p* ≤ 0.01), while males had a higher total attitude score (96.88 ± 15.44 vs. 91.93 ± 13.59, *p* ≤ 0.01). Participants with higher educational levels (secondary and university graduates) had significantly better knowledge (45.91 ± 4.95) and behavior scores (16.52 ± 3.60) than those with primary or preparatory education (42.47 ± 3.47 and 13.89 ± 3.14, respectively, *p* ≤ 0.01). However, participants with lower educational levels had a significantly higher total attitude score (106.84 ± 17.47 for primary vs. 91.21 ± 11.68 for university graduates, *p* ≤ 0.01). Urban residents had higher knowledge (45.61 ± 4.86 vs. 43.40 ± 5.40, *p* ≤ 0.01) and behavior scores (16.46 ± 3.67 vs. 15.24 ± 3.32, *p* ≤ 0.05) compared to rural residents, while rural residents exhibited a higher total attitude score (100.60 ± 18.46 vs. 92.26 ± 13.27, *p* ≤ 0.01). Employed participants showed significantly better knowledge (46.67 ± 4.39) and behavior scores (16.84 ± 3.74) than non-employed participants (44.38 ± 5.16 and 15.92 ± 3.54, *p* ≤ 0.001 and *p* ≤ 0.05). However, non-employed participants had a higher total attitude score (95.31 ± 16.11 vs. 90.61 ± 10.78, *p* ≤ 0.01). Finally, participants who knew someone with mental illness scored higher in knowledge (45.68 ± 5.20 vs. 44.59 ± 4.45, *p* ≤ 0.04) and behavior (17.60 ± 3.32 vs. 13.72 ± 2.78, *p* ≤ 0.01), while those who did not know someone had a higher total attitude score (96.20 ± 12.06 vs. 91.96 ± 15.20, *p* ≤ 0.04) (Table 2).

**Table 2:** The association between the mean score of knowledge, attitude, and behavior towards mental health literacy and the socio-demographic data among the participants, Ain Shams University hospitals, 2022–2023 (*n* = 369)

CAMI subscales	Knowledge	Attitude Dimensions and Total Attitude Score (CAMI)					Behavior
	**MAKS #** **(mean ± SD)**	**AU #** **(mean ± SD)**	**BE #** **(mean ± SD)**	**SR #** **(mean ± SD)**	**CMHI #** **(mean ± SD)**	**Total Attitude Score (CAMI) # (mean ± SD)**	**RIBS #** **(mean ± SD)**
**Socio-demographic data**							
**Age**							
**Adolescents (11–19 years)**	43.16 ± 4.92	25.30 ± 3.93	38.05 ± 4.45	33.94 ± 5.77	25.72 ± 6.35	99.03 ± 17.43	15.53 ± 3.71
**Adults (20 + years)**	46.33 ± 4.69	23.44 ± 3.08	40.33 ± 3.80	35.09 ± 4.06	22.72 ± 4.76	90.74 ± 11.78	16.66 ± 3.56
**t-test**	5.95	4.53	5.09	1.95	4.56	4.69	2.82
***P*-value**	< 0.001**	< 0.001**	< 0.001**	0.05*	0.001**	< 0.001**	0.005**
**Gender**							
**Male**	44.02 ± 5.12	24.94 ± 3.58	38.38 ± 4.75	33.93 ± 4.82	24.22 ± 5.83	96.88 ± 15.44	16.67 ± 3.95
**Female**	45.86 ± 4.83	23.65 ± 3.36	40.12 ± 3.76	35.06 ± 4.61	23.46 ± 5.36	91.93 ± 13.59	16.14 ± 3.50
**t-test**	3.30	3.30	3.74	2.13	1.21	3.05	1.29
***P*-value**	0.001**	0.001**	< 0.001**	0.03*	0.23	0.003**	0.20
**Educational level**							
**Primary**	42.47 ± 3.47	26.47 ± 3.42	36.16 ± 3.48	30.79 ± 4.63	27.32 ± 7.59	106.84 ± 17.47	13.89 ± 3.14
**Preparatory**	41.45 ± 3.73	26.86 ± 3.44	35.64 ± 4.91	31.27 ± 7.62	29.45 ± 7.88	109.41 ± 20.63	13.73 ± 3.63
**Secondary**	44.70 ± 5.04	25.09 ± 4.39	39.74 ± 4.77	34.96 ± 5.32	23.27 ± 5.18	93.42 ± 16.35	17.13 ± 3.29
**University**	45.91 ± 4.95	23.48 ± 3.10	40.12 ± 3.74	35.22 ± 4.05	23.05 ± 4.80	91.21 ± 11.68	16.52 ± 3.60
**One Way ANOVA**	8.52	12.97	13.86	10.20	13.41	19.48	8
***P*-value**	< 0.001**	< 0.001**	< 0.001**	< 0.001**	< 0.001**	< 0.001**	< 0.001**
**Marital status**							
**Never been married**	44.92 ± 5.06	24.26 ± 3.75	39.14 ± 4.29	34.74 ± 5.07	23.95 ± 5.94	94.33 ± 15.81	16.55 ± 3.71
**Ever been married**	46.03 ± 4.77	23.67 ± 2.91	40.25 ± 3.87	34.62 ± 4.06	23.35 ± 4.73	92.22 ± 11.49	15.78 ± 3.49
**t-test**	2.06	1.45	2.47	0.24	0.10	1.46	1.96
***P*-value**	0.04*	0.15	0.01**	0.81	0.32	0.15	0.05*
**Residence**							
**Urban**	45.61 ± 4.86	23.78 ± 3.32	39.78 ± 3.94	35.04 ± 4.47	23.29 ± 5.12	92.26 ± 13.27	16.46 ± 3.67
**Rural**	43.40 ± 5.40	25.62 ± 4.05	38.48 ± 5.22	32.68 ± 5.57	26.20 ± 7.11	100.60 ± 18.46	15.24 ± 3.32
**t-test**	2.95	3.53	1.68	3.36	2.74	3.07	2.22
***P*-value**	0.003**	< 0.001**	0.10	0.001**	0.008**	0.003**	0.03*
**Occupation**							
**Do not work**	44.38 ± 5.16	24.56 ± 3.75	39.06 ± 4.36	34.53 ± 5.20	24.34 ± 6.08	95.31 ± 16.11	15.92 ± 3.54
**Work**	46.67 ± 4.39	23.28 ± 2.88	40.38 ± 3.70	35.01 ± 3.86	22.72 ± 4.36	90.61 ± 10.78	16.84 ± 3.74
**t-test**	4.46	3.69	3.02	1.02	3.00	3.36	2.40
***P*-value**	< 0.001**	< 0.001**	0.003**	0.31	0.003**	0.001**	0.02*
**Knowing someone with a mental illness**							
**No**	44.59 ± 4.45	24.46 ± 3.06	38.71 ± 3.64	33.88 ± 4.33	24.33 ± 5.16	96.20 ± 12.06	13.72 ± 2.78
**Yes**	45.68 ± 5.20	23.82 ± 3.66	40.05 ± 4.32	35.15 ± 4.82	23.35 ± 5.64	91.96 ± 15.20	17.60 ± 3.32
**t-test**	2.08	1.68	2.97	2.48	1.62	2.91	11.86
***P*** **value**	0.04**	0.09	0.003**	0.01**	0.11	0.004**	< 0.001**

*MAK*S Mental Health Knowledge Schedule, *CAMI* Community Attitudes towards Mental Illness, *AU* Authoritarianism, *SR* Social Restrictiveness, *BE* Benevolence, *CMHI* Community Mental Health Ideology, *RIBS* Reported and Intended Behavior Scale. Higher AU scores, lower BE scores, lower SR scores and higher CMHI scores would indicate higher stigma, while the higher values on the total score of attitude scale would indicate higher stigma

(*) Statistically significant at *P* ≤ 0.05, (**) Highly statistically significant at *P* ≤ 0.01

Correlations between knowledge, attitude, and behavior scores among the participants reveal that knowledge (MAKS) was negatively correlated with the total attitude score (r = -0.45, *p* ≤ 0.01), suggesting a moderate negative correlation, where higher knowledge was associated with lower stigma levels. A weak positive correlation was observed between knowledge and behavior (RIBS) (r = 0.29, *p* ≤ 0.001), indicating that greater knowledge was linked to more positive reported and intended behaviors. Behavior, in turn, was moderately negatively correlated with the total attitude score (r = -0.45, *p* ≤ 0.001), reflecting the influence of lower stigma on better behavior outcomes. Among attitude subscales, knowledge showed a moderate positive association with Benevolence (BE) (r = 0.42, *p* ≤ 0.001) and Social Restrictiveness (SR) (r = 0.36, *p* ≤ 0.001) but was moderately negatively correlated with Authoritarianism (AU) (r = -0.35, *p* ≤ 0.001) and Community Mental Health Ideology (CMHI) (r = -0.34, *p* ≤ 0.001). Strong interrelationships were also noted among the attitude dimensions, particularly between CMHI and the total attitude score (r = 0.84, *p* ≤ 0.001), indicating a robust positive correlation, emphasizing their combined impact on behavior (Table 3).

**Table 3:** The correlation between knowledge, attitude, and behavior scores among studied participants, Ain Shams University hospitals, 2022–2023 (*n* = 369)

Variables	Knowledge	Attitude					Behavior
	**MAKS #**	**AU #**	**BE #**	**SR #**	**CMHI #**	**Total Attitude Score (CAMI) #**	**RIBS #**
**Knowledge**							
** MAKS #**							
**r**	1	-0.35	0.42	0.36	-0.33	-0.45	0.29
***P*-value**	-	< 0.001**	< 0.001**	< 0.001**	< 0.001**	< 0.001**	< 0.001**
**Attitude**							
** AU #**							
**r**	-0.35	1	-0.48	-0.54	0.51	-0.31	-0.31
***P*-value**	< 0.001**	-	< 0.001**	< 0.001**	< 0.001**	< 0.001**	< 0.001**
**BE #**							
**r**	0.42	-0.48	1	0.54	-0.49	-0.77	0.34
***P*-value**	< 0.001**	< 0.001**	-	< 0.001**	< 0.001**	< 0.001**	< 0.001**
**SR #**							
**r**	0.36	-0.54	0.54	1	-0.59	-0.84	0.43
***P*-value**	< 0.001**	< 0.001**	< 0.001**	-	< 0.001**	< 0.001**	< 0.001**
**CMHI #**							
**r**	-0.34	0.51	-0.49	-0.59	1	0.84	-0.35
***P*-value**	< 0.001**	< 0.001**	< 0.001**	< 0.001**	-	< 0.001**	< 0.001**
**Total Attitude Score (CAMI) #**							
**r**	-0.45	0.75	-0.77	-0.84	0.84	1	-0.45
***P*-value**	< 0.001**	< 0.001**	< 0.001**	< 0.001**	< 0.001**	-	< 0.001**
**Behavior**							
** RIBS #**							
**r**	0.29	-0.31	0.34	0.43	-0.35	-0.45	1
***P*-value**	< 0.001**	< 0.001**	< 0.001**	< 0.001**	< 0.001**	< 0.001**	-

*r* Correlation Coefficient, *MAKS* Mental Health Knowledge Schedule, *CAMI* Community Attitudes towards Mental Illness, *AU* Authoritarianism, *SR* Social Restrictiveness, *BE* Benevolence, *CMHI* Community Mental Health Ideology, *RIBS* Reported and Intended Behavior Scale. Higher AU scores, lower BE scores, lower SR scores and higher CMHI scores would indicate higher stigma, while the higher values on the total score of attitude scale would indicate higher stigma

(*) Statistically significant at *P* ≤ 0.05, (**) Highly statistically significant at *P* ≤ 0.01

Results of multiple linear regression analyses identifying the predictors of knowledge, attitude, and behavior scores among the studied participants reveal that; For Model 1: Knowledge Score: The model explained 35% of the variance in knowledge scores (*R*^*2*^ = *0.35, p* < 0.001). Higher knowledge scores were significantly associated with being female (β = 0.12, *p* = 0.02), having higher educational levels (university: β = 0.23, *p* = 0.03), living in urban areas (β = -0.12, *p* = 0.01), and being employed (β = 0.21, *p* < 0.001). Age was not a significant predictor (*p* = 0.13). Model 2: Attitude Score: This model accounted for 43% of the variance in attitude scores (*R*^*2*^ = *0.43, p* < 0.001). Lower attitude scores (indicating less stigma) were significantly associated with higher educational levels (secondary: β = -0.30, *p* < 0.001; university: β = -0.47, *p* < 0.001), living in urban areas (β = 0.16, *p* = 0.001), and not knowing someone with a mental disorder (β = -0.11, *p* = 0.02). Age and gender were insignificant predictors (*p* = 0.10 and *p* = 0.17, respectively). Model 3: Behaviour Score: The model explained 59% of the variance in behavior scores (*R*^*2*^ = *0.59, p* < 0.001). Positive behavior scores were significantly associated with being younger (β = -0.19, *p* = 0.01), being female (β = -0.12, *p* = 0.01), having higher educational levels (secondary: β = 0.26, *p* = 0.001; university: β = 0.29, *p* = 0.002), living in urban areas (β = -0.12, *p* = 0.004), being employed (β = 0.12, *p* = 0.01), and knowing someone with a mental disorder (β = 0.50, *p* < 0.001). Across all models, education and residence emerged as consistent predictors, with higher educational levels and urban residence associated with better knowledge, attitudes, and behaviors toward mental health literacy (Table 4).

**Table 4:** Predictors of the MAKS, CAMI, and RIBS scales using multiple linear regression among the studied participants, Ain Shams University hospitals, 2022–2023 (*n* = 369)

Variables	Unstandardized Beta		Standardized Beta	T#	*P*-value
	**B**	**Std. Error**			
**Model 1: Knowledge Score (R**^**2**^ = 0.35, *P*-value = < 0.001**)					
**Age**	-0.05	0.03	-0.10	1.54	0.13
**Gender**	1.29	0.56	0.12	2.30	0.02*
**Education**					
**“Ref. primary”**					
**Preparatory**	-0.88	1.48	-0.04	0.59	0.55
**Secondary**	2.38	1.31	0.16	1.82	0.07
**University**	2.75	1.23	0.23	2.24	0.03*
**Residence**	-1.81	0.73	-0.12	2.48	0.01**
**Occupation**	2.08	0.57	0.21	3.63	< 0.001**
**Model 2: Attitude Score (R**^**2**^ = 0.43, *P*-value = < 0.001**)					
**Age**	0.14	0.08	0.10	1.66	0.10
**Gender**	-2.13	1.55	-0.07	1.38	0.17
**Education**					
**“Ref. primary”**					
**Preparatory**	2.70	4.11	0.05	0.67	0.51
**Secondary**	-13.11	3.64	-0.30	3.60	< 0.001**
**University**	-15.78	3.40	-0.47	4.64	< 0.001**
**Residence**	6.80	2.03	0.16	3.36	0.001**
**Knowing someone with a mental disorder**	-3.42	1.46	-0.11	2.34	0.02*
**Model 3: Behavior Score (R**^**2**^ = 0.59, *P*-value = < 0.001**)					
**Age**	-0.07	0.02	-0.19	3.40	0.01**
**Gender**	-0.95	0.35	-0.12	2.69	0.01**
**Education**					
**“Ref. primary”**					
**Preparatory**	-0.91	0.94	-0.06	0.98	0.33
**Secondary**	2.83	0.83	0.26	3.42	0.001**
**University**	2.49	0.78	0.29	3.21	0.002**
**Residence**	-1.33	0.46	-0.12	2.89	0.004**
**Occupation**	0.92	0.36	0.12	2.55	0.01**
**Knowing someone with a mental disorder**	3.83	0.33	0.50	11.56	< 0.001**

Factors entered in the model are the significant factors in the bivariate analysis; Age and gender were not significant; however, they were added because they are considered universal confounders

(#)T: Represents the t-statistic, which measures the ratio of the difference between the observed and expected values relative to the standard error. It is used to determine the significance of each predictor variable in the regression model. Higher absolute values of T indicate stronger evidence against the null hypothesis

(*) Statistically significant at *p* ≤ 0.05, (**) Highly statistically significant at *p* ≤ 0.01

## Discussion

The societal disapproval associated with psychological disorders remains a significant obstacle for individuals who are affected by it [18]. Based on prior research, it has been observed that developing nations are more prone to harbor stigmatizing attitudes and beliefs towards mental illness as opposed to developed nations [19]. A considerable number of individuals grappling with mental illnesses postpone seeking assistance or consulting a psychiatrist due to their apprehension of being branded as "psychiatric patients" and their desire to evade the detrimental repercussions of societal disapproval [20]. Due to the negative sentiments directed toward their affliction, individuals with mental illnesses endure a decline in their overall quality of life [21].

The present study uncovered a statistically significant disparity between adults and adolescents in terms of their knowledge, attitude, and behavior concerning mental health literacy. Specifically, adults exhibited superior knowledge and behavior in relation to mental health disorders, while adolescents displayed a more favorable attitude compared to adults. These findings contradict the findings of *Louise *et al*., 2008* [22], who noted that, although mental health literacy pertaining to schizophrenia and depression varied across different age groups in the Australian community, younger individuals possessed greater knowledge about the diagnosis and treatment of depression than older individuals. Furthermore, Louise added that younger individuals’ mental health literacy regarding schizophrenia is not as advanced as that of depression. The researchers ascribed the lower mental health literacy among older individuals to a range of factors, including the circumstances in which older individuals were less inclined to access media-oriented mental health messages (such as the Internet). The primary source of mental health literacy for younger individuals, that is, educational programs provided by schools, was not commonly accessible for adults in preceding decades. Older adults were not the intended audience for the ongoing mental health literacy campaigns. The cause for the discrepancy between the current findings and results of Louise et al. [22] could be attributed to the distinct techniques of age stratification employed in each study. Moreover, personal encounters with patients suffering from mental illness have a noteworthy influence on mental health literacy in Egypt, and these encounters become more widespread as participants grow older.

In terms of knowledge and attitude towards mental health literacy, there was a statistically significant difference between the male and female populations; the male population exhibited a more positive attitude towards mental health, while the female population showed better response in terms of understanding mental health disorders.

Within Western societies, it is widely acknowledged that women were more knowledgeable than males [23–27]. Yet, in certain cultural contexts, this gender effect might, however, be the opposite. A cross-sectional survey conducted in Qatar, discovered that men exhibited superior knowledge, attitudes, and beliefs regarding mental health when compared to women [28]. This suggests that cultural influences may be a factor in this variation.

The findings of the present study demonstrated that individuals with lower levels of education exhibited a markedly more positive attitude towards mental health disorders, whereas those with higher educational attainment demonstrated significantly greater knowledge and better behavior concerning mental health illness. A study in Portugal further strengthens this finding, as the authors stated that among diverse groups of individuals residing in Póvoa de Varzim, a municipality located in the northern region of Portugal, MHL increases in line with higher educational achievement. The authors consistently observed that individuals with higher levels of education exhibit reduced stigma towards people struggling with mental health illness. This phenomenon can be explained by the fact that education can help people become more aware of and knowledgeable about mental health issues, which can help to reduce stigmatizing attitudes and the fear and misinformation that often accompany them [29]. Education can also expose people to a greater variety of viewpoints and experiences, which can promote empathy and lessen the propensity to use stereotypes or judge other people [30].

The present investigation demonstrated that despite rural inhabitants possessing a more favorable attitude towards mental health disorders, urban dwellers exhibited significantly greater knowledge and better behavior related to mental health disorders. This outcome aligns with the observations made by a study conducted in the USA [31], which remarked that geographical location can impact stigma levels. Individuals with mental illnesses residing in rural areas may experience more stigma in comparison to their urban counterparts due to their condition. Moreover, there exists a correlation between stigma and mental health literacy, with stigma decreasing as mental health literacy increases.

The outcomes of the present investigation indicate that individuals who are employed possess significantly greater understanding and better behaviors pertaining to mental health disorders, whereas individuals who are retired or unemployed exhibit a more favorable attitude towards these conditions. These findings align with those of a German study, which revealed that individuals without employment generally exhibited slightly higher health literacy scores compared to their employed counterparts. The discrepancy between the two studies may be attributed to variations in the definitions and measurements of mental health literacy, as well as differences in sample populations and contexts. Factors such as age, gender, culture, and exposure to mental health information and services likely influence these variations, shaping the mental health literacy levels of the respective groups [32].

According to the findings of the current study, individuals who possess an acquaintance affected by a mental disorder demonstrate a significantly greater extent of awareness and behavior regarding mental illness, whereas those lacking an association with an individual suffering from a mental disorder exhibit superior attitudes regarding mental illness. This result is consistent with the study carried out in Portugal [33], which determined that living close to someone who has a mental illness improves mental health literacy and reduces the stigma attached to mental illness. Numerous research works have proposed that social interaction with individuals who suffer from mental illness can significantly reduce stigma [34–36]. The results of the study showed that knowledge (MAKS) was negatively correlated with the total attitude score, suggesting that higher knowledge was associated with lower stigma levels. Positive correlations were observed between knowledge and behavior (RIBS), indicating that greater knowledge was linked to more positive reported and intended behaviors. Behavior, in turn, was negatively correlated with the total attitude score, reflecting the influence of lower stigma on better behavior outcomes.

These findings are consistent with several studies [3, 34, 37–42] that have similarly reported that greater knowledge of mental health issues correlates with more positive attitudes and understanding and lower stigma levels. However, some studies [43, 44] reported opposing results, where increased knowledge leads to distancing from individuals with mental health conditions. This counterintuitive finding suggests that while people may become more informed about the symptoms and behaviors associated with mental illness, this awareness can also foster discomfort or fear, contributing to social distancing. Thus, the findings underscore the complex relationship between knowledge and attitudes towards mental illness. While increased knowledge can lead to improved understanding and empathy for those affected, it can also contribute to negative perceptions and social distancing if individuals focus primarily on the symptoms of the illness rather than the person. This highlights the need for a nuanced approach in mental health education, which not only increases factual knowledge but also addresses misconceptions and promotes more inclusive, supportive behaviors towards individuals with mental health conditions.

Raising public awareness of mental health issues is vital, however; there might be unintended consequences as well, such as a rise in stigma or avoidance of those who struggle with mental illness. Consequently, people who require assistance and care may experience stigma, discrimination, and social isolation. It is crucial to develop and execute mental health education programs that can foster acceptance and empathy while lowering fear from people living with mental illness.

### Study limitations

This study’s cross-sectional design limits the ability to draw causal inferences, as it captures only associations at a single point in time. Additionally, the time required to complete the instruments used in the study may have led to respondent fatigue, potentially influencing the accuracy of the responses. The self-reported nature of the questionnaire responses also introduces the possibility of information bias, as participants may have been influenced by personal perceptions or social desirability when answering the questions. Furthermore, the study did not achieve an equal or near-equal sample size between adolescents and adults, which could affect the generalizability of the results. Ensuring a balanced representation across age groups would help to minimize this bias and provide more reliable and broadly applicable conclusions.

## Conclusion

The study highlights the complex interplay between socio-demographic factors and mental health literacy components—knowledge, attitude, and behavior—demonstrating the pivotal roles of education, urban residency, and familiarity with mental illness. Adults had better knowledge and behavior, while adolescents showed more favorable attitudes. Education and urban residency emerged as consistent predictors of improved mental health literacy, underscoring the need for targeted interventions. Recommendations include integrating mental health education into curricula, community awareness campaigns to reduce stigma, workplace training programs, and improved rural access to mental health services. Longitudinal research and Key performance indicators (KPI) based evaluations are essential to sustaining these efforts and enhancing outcomes across diverse populations.

## Data Availability

Data are available from the corresponding author upon request.

## Abbreviations

MAKS: Mental Health Knowledge Schedule
CAMI: Community Attitudes towards Mental Illness
RIBS: Reported and Intended Behavior Scale
AU: Authoritarianism
SR: Social Restrictiveness
BE: Benevolence
CMHI: Community Mental Health Ideology
KPI: Key Performance Indicators
